# Halogen bonds between ligands and proteins: Can we use them in validation?

**DOI:** 10.1002/pro.70321

**Published:** 2025-10-11

**Authors:** Ida de Vries, Georgia Tsiompanaki, Anastassis Perrakis, Robbie P. Joosten

**Affiliations:** ^1^ Department of Biochemistry Oncode Institute and The Netherlands Cancer Institute Amsterdam The Netherlands

**Keywords:** halogen bonds, halogen‐π interactions, ligand validation, ligands, protein structure

## Abstract

Halogen bonds are polar interactions between a halogen atom and its acceptor. They have characteristic geometry induced by the positively charged σ‐hole on the halogen atom. Despite their importance in drug development, halogen bonds are often overlooked in the analysis and validation of ligand‐protein complexes. We analyzed halogen bonds between ligands and proteins in structure models from the PDB‐REDO databank and defined key geometric parameters. These are the donor‐acceptor distance and two bond angles (θ1 and θ2) for interatomic halogen bonds, or in the case of halogen‐π interactions, the distance between the halogen and acceptor π‐system and a bond angle (θ1). Based on the distribution of these geometric parameters, we introduce a score, HalBS, that marks whether halogen bonds are adopting the preferred or allowed geometry or are an outlier and should be examined critically. A reference implementation of this score is now available in PDB‐REDO and the source code at https://github.com/PDB-REDO/HalBS. This is a first step towards improving halogen bond treatment in the analysis of macromolecular structure models.

## 1 INTRODUCTION

**FIGURE 1:**
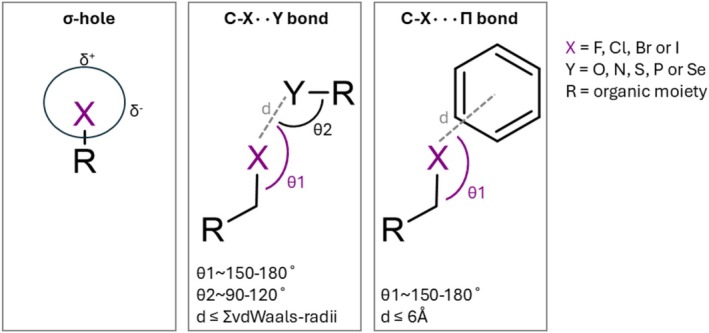
Schematic representation of the σ‐hole on a halogen atom (left), and halogen bond characteristics of C‐X··Y halogen bonds (middle) and C‐X···Π halogen bonds (right).

Halogen bonds, sometimes referred to as X‐bonds, are polar interactions between a halogen atom and an acceptor atom. A halogen bond occurs due to polarization of the σ‐bond between the halogen and its connecting atom. This causes a positive charge on the opposite side of the halogen atom, the so‐called σ‐hole (Figure 1; Clark et al., 2007). As a result, the acceptor of the halogen bond is typically an atom with a free lone pair, like an oxygen, nitrogen, sulfur, phosphorus, or selenium atom. Additionally, halogen atoms can interact with π‐systems (Bent, 1968). Due to differences in polarization properties, halogen bonds with iodine are the strongest, followed by bromine, chlorine, and finally halogen bonds with a fluorine atom are the weakest halogen bonds (Cavallo et al., 2016; Gilday et al., 2015; Politzer et al., 2010).

Halogen bonds can be characterized based on key geometric parameters. For the “regular” interatomic halogen bond, in this study referred to as C‐X··Y halogen bonds, the first parameter is the distance (d) between the halogen and acceptor atom. Halogen bonds are longer than hydrogen bonds and weaker because their Pauli repulsion is larger. Nevertheless, the polarized, electrostatic force between the σ‐hole of the halogen atom and the free electrons of the acceptor atoms causes orbital overlap and charge transfer effects (Huber et al., 2013; Margiotta et al., 2020). This can result in a shorter interatomic distance than the sum of the Van der Waals radii of the atoms (Auffinger et al., 2004; Hassel, 1970).

For the orientation of the acceptor with respect to the halogen atom, two angles are defined: θ1, the angle between the halogen‐containing compound and the acceptor, and θ2, the angle between the halogen atom and the acceptor‐containing compound (Figure 1). These angles show distinct ranges, caused by the position of the σ‐hole of the halogen atom. For C‐X··Y halogen bonds, ideally θ1 spans between ~150° and ~180° and θ2 between ~90° and ~120° (Bent, 1968; Cavallo et al., 2016; Desiraju et al., 2013; Scholfield et al., 2013; Turunen et al., 2021). In the case of halogen bonds between a halogen atom and a π‐system, the halogen–π interaction, in this study referred to as C‐X···Π halogen bonds (Figure 1), only the distance between the halogen atom and the π‐system, and the θ1 angle are defined (Cavallo et al., 2016; Desiraju et al., 2013; Jubb et al., 2017; Kellett et al., 2020).

Halogen atoms and their potential to form halogen bonds are used in drug design, as halogens affect the affinity, selectivity, and efficacy of therapeutics (Fraley & Sherman, 2018; Kortagere et al., 2008; Lu et al., 2009; Lu et al., 2010). Halogen substituents increase the overall hydrophobicity of a ligand, thereby enhancing the hydrophobic effect of binding, driven by the minimization of unfavorable water–nonpolar contacts upon binding, and yielding a favorable entropic contribution to the binding free energy (Langton et al., 2016). Thus, halogenation of ligands alters the conventional hydrophobic effect by combining the nonspecific entropic gain of water release with the specific directional interactions inherent to halogen bonds (Verteramo et al., 2024; Wang et al., 2019). While fluorine atoms make the weakest halogen bonds among their peers, they are the most used halogen atoms in drug design. Due to their high bond strength and small atom size compared to the other halogen atoms, drug molecules involving fluorine are less prone to metabolic toxicity and fit in smaller protein binding pockets (Henary et al., 2024; Shah & Westwell, 2007).

These electrostatic and geometrical unique properties make halogen atoms attractive features in drug design but also in the earlier, more explorative drug lead discovery phase. Herein, halogens are often found in molecules used in virtual and fragment screening libraries, used to discover potential new binders to a target protein (Chopra et al., 2023; Dammann et al., 2022; Heidrich et al., 2019). Particularly, libraries that include fluorine‐containing compounds are especially valuable in fragment screening using Nuclear Magnetic Resonance (NMR) (Li & Kang, 2024; Troelsen et al., 2020). Fluorine atoms are not abundant in endogenous molecules but are favorable for NMR due to their high sensitivity and wide chemical shift range (Buchholz & Pomerantz, 2021). Hence, fragment screening NMR is typically used to discover protein binding sites and protein dynamics (Danielson & Falke, 1996; Li & Kang, 2024). They can also be used to identify compounds that bind to RNA (Kreutz et al., 2006).

Despite their overall benefits for drug design and discovery, halogen bonds are often not included in macromolecular structure model validation (Zhang et al., 2017). Historically, macromolecular validation pipelines and software, like PROCHECK (Laskowski et al., 1993), WHAT_CHECK (Hooft, Vriend, et al., 1996), and MolProbity (Chen et al., 2010), have been designed to validate the geometries of proteins and nucleic acids (Read et al., 2011). The description of ligands and the interactions they make is typically less well considered, or only more recently (Read et al., 2011; Ceruto‐Massagué et al., 2013; Adams et al., 2016; Gore et al., 2017; Smart et al., 2018). One of the few places where halogen bonds are explicitly considered, is at the PDBe‐KB (Protein Data Bank in Europe Knowledge Base) (Choudhary et al., 2025; PDBe‐KB consortium, 2022) pages for ligands, where the detection of halogen bonds originates from Arpeggio (Jubb et al., 2017). Such halogen bond determination, for example, explicitly highlights the differences in interactions between T_3_ (3,5,3′‐triiodo‐l‐thyronine) and T_4_ (3,5,3′,5′‐tetraiodo‐l‐thyronine) hormone binding to the thyroxine‐thyroid hormone receptor (Sandler et al., 2004). T_4_ has an iodine atom at the 5′ position of a benzene ring, which is not present in T_3_. Inspection of the ligand interactions on the PDBe ligand pages of the corresponding protein‐ligand models (PDB‐ID: 1Y0X (T_4_) and 1XZX (T_3_)) shows that this 5′‐iodine interacts with the receptor, which demonstrates the importance of halogens in protein modulation and thus in drug discovery.

Here, we analyze halogen bonds found in PDB‐REDO structure models and aim to enrich the PDB‐REDO validation tools with a metric for halogen bonding interactions. We propose such a metric for interactions between halogen‐containing ligands and amino acids. Hereby, halogen bonds can be incorporated and integrated into pipelines that validate protein‐ligand interactions, which we demonstrate for PDB‐REDO.

## 2 RESULTS AND DISCUSSION

### 2.1 Defining halogen bonds between ligands and proteins in PDB‐REDO

To define halogen bonds in macromolecular structure models, we selected every fluorine (F), chlorine (Cl), bromine (Br), and iodine (I) atom present in PDB‐REDO structure models. These were stored together with their most likely acceptor atoms (i.e., O, N, S, P, or Se). We used the structure models from the PDB‐REDO databank to ensure all models are uniformly treated and no specific restraints for halogen bond interactions were used (Joosten et al., 2009). Note that particularly the heavier halogen atoms are prone to radiation damage, which leads to higher B‐factors (Garman & Weik, 2019; Rodrigues et al., 2024). To ensure high‐quality data, while retaining as many datapoints as possible, we rejected halogen atoms with a B‐factor >100 Å^2^, Real Space Correlation Coefficient (RSCC; Jones et al., 1991) for the compounds containing the halogen or acceptor atom below 0.9, or which were in structure models with a resolution worse than 2.5 Å (complete distributions in Figure S1).

Halogen bonds can be formed with different acceptor atoms and residues in proteins. Backbone oxygen atoms, but also oxygen atoms in the side chains of amino acids, can serve as acceptor atoms. Furthermore, nitrogen atoms with a free lone pair and sulfur atoms in amino acid side chains can fulfill this role (Wilcken et al., 2013). Regarding the nitrogen atom as an acceptor, only histidine residues are, depending on the context‐specific protonation state, potential acceptors within a protein. However, within our initial data, we did not find high‐confidence observations; hence, nitrogen atoms were excluded for the remainder of this study.

To be able to differentiate between acceptors for C‐X··Y bonds in macromolecular structure models, we mined the acceptor with its neighboring connecting atom. For example, if the acceptor is an oxygen atom of a hydroxyl group on a carbon atom, the carbon atom is stored with the oxygen atom and the acceptor is marked as ‘O‐C'. The acceptors of the found halogen bonds mainly appear to be O‐C acceptors, followed by S‐C. There are a few observations for acceptor atoms attached to a non‐carbon atom (Table S1), which, for example, occurs when the acceptor is a non‐canonical amino acid. These were too few observations to define sensible validation targets for these acceptors. Therefore, only O‐C and S‐C acceptors were finally selected for further analysis of C‐X··Y bonds.

For the halogen bonds involving a π‐system, the side chains of histidine, phenylalanine, tyrosine, and tryptophan are acceptor candidates (Wilcken et al., 2013). We mined these to identify C‐X···Π halogen‐acceptor pairs in PDB‐REDO structure models. Halogen bonding interactions with the π‐system of phenylalanine are found most (3481), followed by tyrosine (2442), histidine (1549), and tryptophan (935) (Table S1). We then selected only the halogen bonds in which the halogen atom is in a ligand, that is, any compound marked as ‘non‐polymer’ in the CCD, (Table S2) and the acceptor is a canonical amino acid in a protein.

The final dataset contains 8423 C‐X··Y and 8096 C‐X···Π ligand‐protein halogen bonding interactions (Table 1), which were used in downstream analyses.

**TABLE 1:** Number of observations of halogen‐acceptor pairs of ligand‐protein interactions for C‐X··Y and C‐X···Π bonds found in PDB‐REDO structure models.

Number of halogen‐acceptor pairs in ligand‐protein interactions					
Halogen acceptor	F	Cl	Br	I	Total
	C‐X··Y halogen bonds				8423
O‐C	4381	456	2479	156	7472
S‐C	554	80	312	5	951
	C‐X···Π halogen bonds				8096
Phenylalanine	2097	167	1100	49	3413
Tyrosine	1328	102	895	14	2339
Histidine	1135	55	257	9	1456
Tryptophan	539	121	225	3	888

*Note*: For C‐X··Y bonds, the acceptor is listed with its neighboring atom, e.g., O‐C is an oxygen atom as an acceptor connected to a carbon atom. Accepting π‐systems are listed by amino acid.

### 2.2 The geometry of ligand‐protein halogen bonds in PDB‐REDO

**FIGURE 2:**
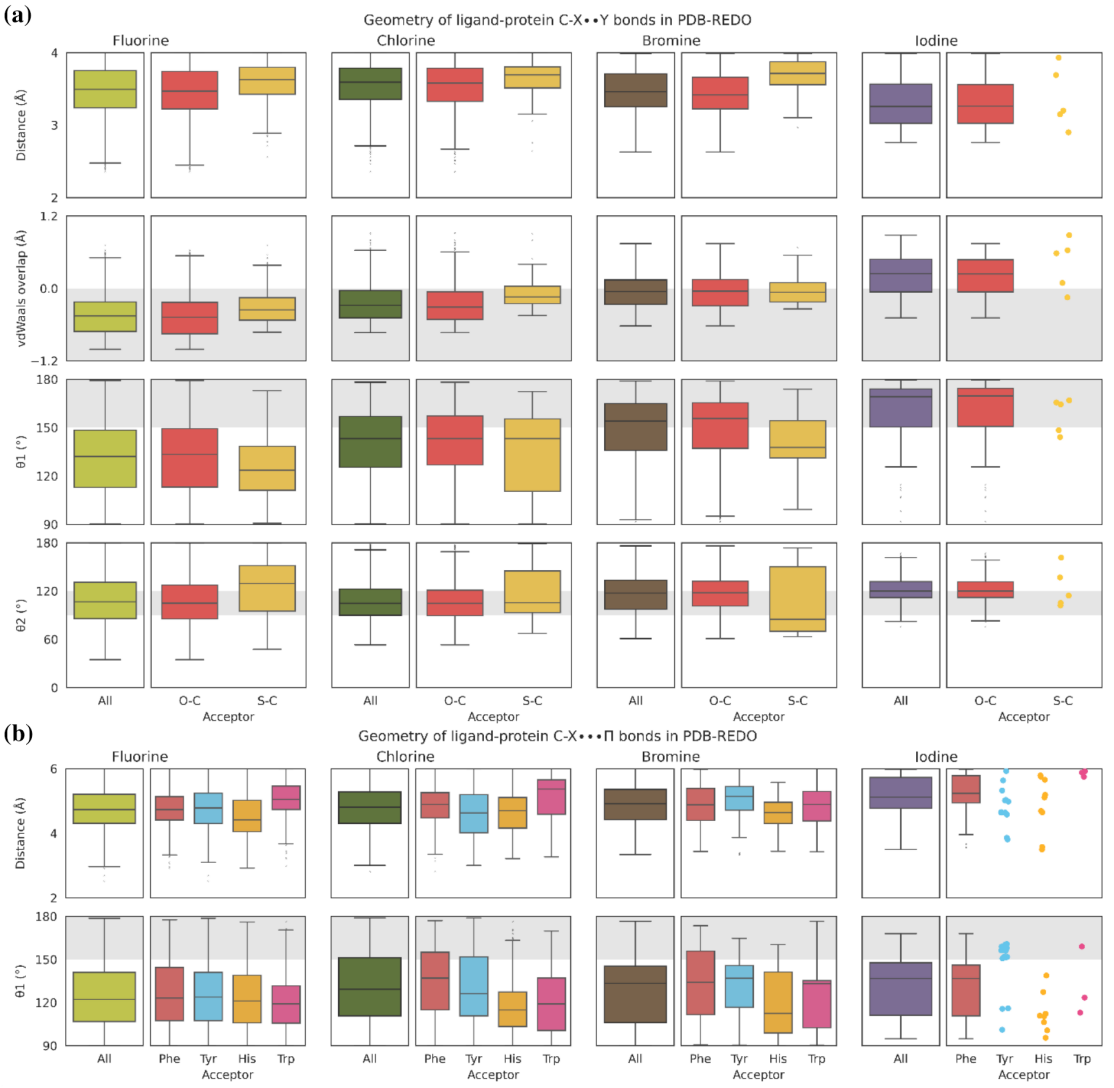
(a) Geometry of ligand‐protein C‐X··Y halogen bonds found in PDB‐REDO. For each halogen (from left to right: Fluorine (light green), chlorine (dark green), bromine (brown), iodine (purple)) the overall distance, Van der Waals overlap, θ1 and θ2 angles are shown on the left panel. In the panels on the right‐hand side these values are split per acceptor: Oxygen (red, O‐C), and Sulfur (yellow, S‐C). Note that a positive value for the Van der Waals overlap means that the distance between halogen and acceptor atom is shorter than sum of the Van der Waals radii. (b) Geometry of ligand‐protein C‐X···Π halogen bonds found in PDB‐REDO. For each halogen (from left to right: Fluorine (light green), chlorine (dark green), bromine (brown), iodine (purple)) the overall distance and θ1 angle are shown on the left panel. In the panels on the right‐hand side these values are split by acceptor: The π‐system in the side chains of histidine (yellow), phenylalanine (red), tyrosine (blue) and tryptophan (pink). Gray areas indicate the expected ranges of the geometries. Boxes span from first to third quartile, the median is depicted as the middle line and whiskers extend to 1.5 times the interquartile range (IQR). Statistical values corresponding to the boxplots (number of observations, mean, median, Q1, Q3 and Median absolute deviation (MAD)) can be found in Tables S3 and S4 for panels A and B, respectively. For clarity a strip plot is created when there are 25 or fewer observations.

For the ligand‐protein halogen bonds in the dataset, we calculated the characteristic halogen bond geometries: distance, Van der Waals overlap, and the θ1 and θ2 angles for C‐X··Y halogen bonds; and distance and θ1 angle for C‐X···Π halogen bonds. We then investigated the distribution of these metrics and visualized them as boxplots (Krzywinski & Altman, 2014), unless there were 25 or fewer observations, in which case individual observations are shown (Figure 2).

From the boxplots for C‐X··Y halogen bonds (Figure 2a), we observe that the distribution of the distance of the halogen bond is rather narrow (around 3.5 Å), independent of halogen type or acceptor atom. Note that the median distance becomes shorter and the distribution broader for halogen bonds involving iodine. A Van der Waals overlap between the halogen atom and its acceptor is barely present for fluorine and chlorine. Notably, for bromine and even more so for iodine, halogen‐acceptor pairs do show an overlap with their Van der Waals radii. When investigating the θ1 angle for protein‐ligand C‐X··Y halogen bonds, we observe smaller median angles than the expected range of 150°–180°. Particularly, halogen‐acceptor pairs involving fluorine (132°) and chlorine (143°) have smaller median θ1 angles, while for bromine (154°) and iodine, the median θ1 angle (169°) is within the expected range. Contrary to the θ1 angles, the median θ2 angles are within their expected range of 90°–120°, independent of the halogen type.

For C‐X···Π halogen bonds (Figure 2b), we observe that the median distance of the halogen bond is rather stable (around 4.8 Å) for fluorine, chlorine, and bromine, while the halogen bonds involving iodine are longer (median 5.1 Å). The observed θ1 angles for the halogen‐π bonds have a median around 110°–140°, whereas we would expect them to range between 150° and 180°. While the distances between halogen and acceptor π‐system are not substantially different for different acceptor amino acids, the θ1 angles show different preferences. Particularly, the histidine and tryptophan residues seem to have a smaller θ1 angle in halogen bonds with chlorine and bromine.

### 2.3 Validation of halogen bonds

For the C‐X··Y halogen bonds, we observe increasing Van der Waals overlap for halogen‐acceptor pairs with increasing halogen atom size (fluorine < chlorine < bromine < iodine). Following a similar trend, the θ1 and θ2 angles reach their expected values with increasing halogen atom size. This fits the increasing polarization of the σ‐hole and the decreasing electronegativity of halogen atoms (Cavallo et al., 2016; Politzer et al., 2010), resulting in the strongest halogen bonds involving iodine and weakest involving fluorine atoms as the Van der Waals overlap becomes more pronounced when halogen atom size increases. Hence, halogen interaction pairs involving fluorine or chlorine should be interpreted with care and might not be considered ‘true’ halogen bonds. On the other hand, halogen‐acceptor pairs with bromine and iodine are more in line with theory and hence can be considered ‘true’ halogen bonds. Note that C‐X··Y halogen bonds involving bromine show little Van der Waals overlap, but the θ1 and θ2 angles are within the expected ranges. Halogen bonds involving iodine are even more in line with “textbook” halogen bonds: they show Van der Waals overlap, their θ1 angle nicely ranges between 150° and 180°, and the θ2 angles also obey their theoretic values by averaging around 120°.

Note that the θ1 angle in C‐X···Π halogen bonds is on average 127°, which is substantially smaller than the expected 150° to 180° range (Figure 2b). This trend is independent of the type of halogen atom.

As no restraints were applied on the θ1 and θ2 angles in the PDB‐REDO model generation, these angles are unbiased. The Van der Waals overlap is weakly restrained compared to the distances, so the bias for the Van der Waals overlap is considered to be minimal. Furthermore, our data quality is sufficient for analysis of distributions (Figures S2 and S3). Therefore, we proceed with defining target values for all halogen bond categories, except C‐I···Π halogen bonds involving tryptophan, as there are fewer than five observations for these cases. We define a way to judge the quality of halogen‐acceptor pairs while considering that the distributions of the metrics regarding the geometry are mono‐disperse, but not normally distributed (Figures S4 and S5). Hence, it is not prudent to determine targets as means with standard deviations for a halogen bond.

We evaluate the geometric quality of a potential halogen bond by calculating a score for each relevant geometric parameter, referred to as *Score*_*geom*_, using the following equation:

(1) $${\text{Score}}_{\text{geom}}=\max\left[\;\frac{\text{value}-Q3}{\mathrm{IQR}},\frac{Q1-\text{value}}{\mathrm{IQR}},0\right]$$

Here, *value* is the measured value of a geometric parameter; *Q1* and *Q3* are the first and third quartiles, respectively; and *IQR* is the interquartile range (Q3 − Q1), derived from the boxplot statistics of the applicable reference dataset. The score expresses the distance of a value from the boundaries of the ‘box’ of the reference dataset in terms of the number of IQRs. Taken that a value within Q1–Q3 (i.e., the interquartile range) is considered preferred, a value within 1.5 × IQR of the quartiles (i.e., within the whiskers) is allowed, and a value outside the whiskers is treated as an outlier, this means that any outlier will have a *Score*_*geom*_ >1.5.

We then apply Equation (1) independently to each geometric parameter relevant to the halogen bond geometry. For C–X··Y interactions, *Score*_*geom*_ is computed for distance, θ1, and θ2; and for C–X···Π interactions, it is computed for distance and θ1 only, as θ2 is not defined in this context. We then define an overall Halogen Bond Score (HalBS) as the maximum of the individual geometric scores:

(2) $$\text{HalBS}=\left\{\begin{array}{c} \;\max\left[{\text{Score}}_{\text{distance}},{\text{Score}}_{\theta 1},{\text{Score}}_{\theta 2}\right],\;\text{if}\;\theta 2\;\text{is defined}\\ \max\left[{\text{Score}}_{\text{distance}},{\text{Score}}_{\theta 1}\right],\;\text{otherwise} \end{array}\right\}$$

This score reflects the least favorable geometric feature of the halogen bond and is used as a proxy for assessing bond quality. A HalBS >1.5 indicates that at least one geometric parameter is an outlier, suggesting that the halogen bond geometry may be abnormal and should be examined more closely.

The robustness and accuracy of HalBS improve with the quantity and diversity of data used to compute the reference boxplot statistics. For example, in C–X···Π interactions involving amino acids like tyrosine, histidine, or tryptophan, limited available data may affect the reliability of the scoring.

### 2.4 HalBS implementation in PDB‐REDO

A reference implementation of HalBS reports scores for C‐X··O, C‐X··S, C‐X···His, C‐X···Phe, C‐X···Tyr, and C‐X···Trp halogen bonds where X = F, Cl, Br, I, with the exception of C‐I···Trp, for which boxplot statistics could not be calculated due to insufficient data (Table S4). Halogen atoms in alternate conformations are excluded, as well as water molecules functioning as potential acceptors. Furthermore, the θ1 angle between the halogen atom and its potential acceptor must be larger than 90°. When multiple halogen bonds are possible for a single halogen atom (C‐X··Y and C‐X···Π combined), the bond with the lowest HalBS score is selected and reported. Note that while the boxplots were generated for halogen‐containing ligands, the implementation of HalBS is calculated for any type of halogen‐containing compound, including non‐canonical amino acids and nucleotides. The implementation is added to PDB‐REDO (version 8.16), which now reports the number of halogen bonds and the mean HalBS for each structure model in mmCIF (Westbrook et al., 2022) format. Hereby, we aim to increase awareness and create more data to improve the halogen bond definitions and validation pipelines in the future. PDB‐REDO entries with detected halogen bonds can be retrieved through the PDB‐REDO archive manager at https://pdb-redo.eu/archive/.

### 2.5 Example cases

**FIGURE 3:**
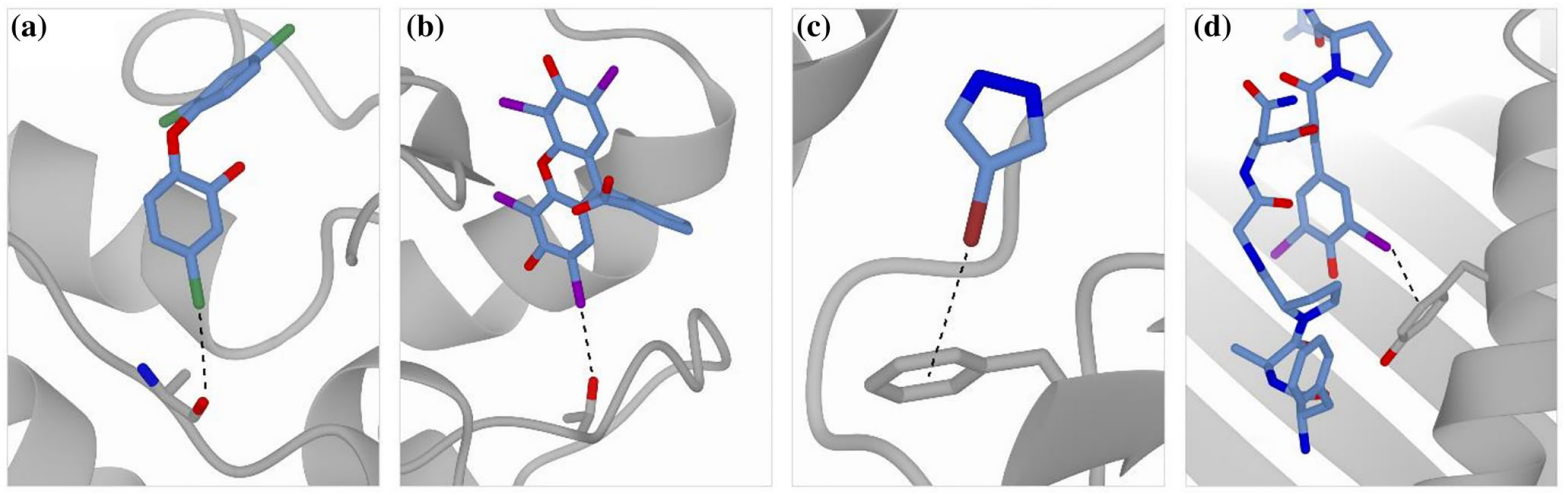
Examples of halogen bonds (marked as a black dotted lines). A) C‐X··Y halogen bond between chlorine of triclosan (atom CL14 of TCL A371) and the carbonyl oxygen atom of alanine A97 (PDB‐ID: 3GR6, HalBS: 0.36. B) C‐X··Y halogen bond between iodine of erythrosine extra bluish (atom I of 9ZZ A303) and hydroxylic oxygen of threonine A77 (PDB‐ID 5OOH, HalBS: 1.63. C) C‐X···Π halogen bond between bromine atom BR4 of BYZ B201 with the π‐system of phenylalanine B154 (PDB‐ID: 8CZM, HalBS: 0.07) D) C‐X···Π halogen bond between iodine (atom I1 of TYI C6) with the π‐system of tyrosine A159 (PDB‐ID 4PGC, HalBS: 0.90).

The binding of the anti‐microbial agent triclosan (TCS) to its target protein enoyl‐[acyl‐carrier‐protein] reductase (ENR), one of the proteins involved in fatty acid synthesis in bacteria, causes ENR to change to a closed conformational state, as seen in PDB entry 3GR6 (Priyadarshi et al., 2010). Besides the described electrostatic interactions between the binding pocket and triclosan, the inhibitor forms a halogen bond with its chlorine close to the carbonyl oxygen atom of the Ala97 backbone (Figure 3a). Notably, this alanine is in the loop that is involved in the conformational change suggesting that the halogen bond contributes to the change in conformation of the loop in ENR.

An example where halogen bonds play a role in drug development is erythrosin extra bluish, which was found to be a hit compound in a study targeting biliverdin reductases type B (BLVRB) (Nesbitt et al., 2018). In PDB entry 5OOH we observe that the compound has multiple interactions with the protein, among which is the iodine atom that is forming a C‐X··Y halogen bond (Figure 3b).

Halogen atoms often appear in crystallographic fragment screening experiments, where multiple small organic compounds are screened towards a target protein. Bacterial oxidoreductase in *E. coli* DsbA (EcDsbA) is such a target. It is responsible for the formation of disulfide bonds in many bacterial factors causing disease, and hence is considered a target in the treatment of drug‐resistant bacterial infections (Whitehouse et al., 2023). In PDB entry 8CZM, one of the bromine‐containing fragments is BYZ (4‐bromo‐1H‐pyrazole), which binds to EcDsbA and forms a C‐X···Π halogen bond with the π‐system of phenylalanine in the protein (Figure 3c).

An example of a modified amino acid containing halogens is 3,5‐diiodotyrosine (TYI), which was used to investigate peptide selectivity in the major histocompatibility complex (MHC) class I (PDB‐ID 4PGC) (Garstka et al., 2015). In this study, increasingly larger residues were used to probe the peptide binding site of MHC class I. Although the iodine atoms were used for their size, one iodine makes a clear C‐X···Π halogen bond with a tyrosine in the binding site (Figure 3d).

The examples described above demonstrate perfectly fine halogen bonds according to theory. The halogen bonds involving triclosan, the BYZ fragment, and the modified amino acid TYI (Figure 3a, c, d) all have a HalBS < 1.5, indicating a preferred halogen bond geometry. However, the halogen bond involving erythrosine extra bluish (Figure 3b) adopts a score >1.5, suggesting it should be considered with caution. We suggest that the current HalBS cannot be used as a direct validation metric but can provide an indication of genuine halogen bonds and “not so proper” halogen bonds.

## 3 FUTURE CONSIDERATIONS

To further optimize validation of C‐X··Y halogen bonds, it could be investigated whether different types of halogen bond geometries are possible (Ibrahim et al., 2022). Separating C‐X··O halogen bonds into carbonyl oxygen, carboxyl, and hydroxyl oxygen atoms might improve the accuracy of halogen bond descriptions (Figure S6) (Costa, 2017; Zhang et al., 2017). Another challenge is to identify C‐X··N halogen bonds with histidine as the acceptor. The histidine protonation state defines whether such interaction is possible, but depending on the local molecular context, reliably establishing this can range from straightforward to computationally expensive (Hooft, et al., 1996; Evans & Murshudov, 2013). Besides forming C‐X··N halogen bonds, histidine residues can (depending on the protonation state) also form hydrogen bonds with halogen atoms, mainly involving highly electronegative fluorine atoms (Pietruś et al., 2022). In ligand‐protein complexes, such bonds can involve the histidine side chain or the hydrogen atom present in the peptide bond of a protein backbone. We have not examined hydrogen bonds between a halogen and a hydrogen atom in this study. Limitations are that typically the resolution of X‐ray crystallography is not good enough to determine the exact position of a hydrogen atom, and the protonation state of the halogen neighborhood is not always defined properly. This could be calculated using, for exampl, QM/MM (quantum mechanics/molecular mechanics) calculations, but these require expertise and can become expensive depending on the molecular context (Hooft, et al., 1996; Evans & Murshudov, 2013). Hence, we concluded they are not feasible at the scale of ligand validation in PDB‐REDO.

Arpeggio (Jubb et al., 2017) is a software that uses a slightly different approach to define halogen bonds. A Structural Interaction Fingerprint (SIFt) (Deng et al., 2004) of a ligand binding site is determined first, and its interactions are then defined in 3‐dimensional space. A halogen bond is defined as an interaction between a halogen atom (F, Cl, Br, or I) and a donor atom when θ1 is 120°, θ2 spans between 70° and 170°, and the ideal distance is 1.85 Å (PDBe Europe, n.d.). Arpeggio also defines weak halogen bonds, wherein the Van der Waals radius of the halogen atom plus the Van der Waals radius of a hydrogen atom and a compensating factor are considered additionally to θ1 being between 30° and 150° (PDBe Europe). As Arpeggio is integrated in the PDBe ligand pages (Choudhary et al., 2025; PDBe‐KB consortium, 2022), we compared its results with our approach for the example cases in Figure 3. Arpeggio identifies the halogen bond between triclosan and ENR (Figure 3a). However, it defines a weak interaction with the backbone nitrogen atom of Ala 97, whereas we define the interaction with the backbone carboxyl oxygen of the same residue. Arpeggio also identifies the halogen bond between erythrosin extra bluish and BLVRB (Figure 3b), and the halogen‐π interaction between BYZ and EcDsbA (Figure 3c). Note that the definition of the latter is defined based on the distances to all carbon atoms of the phenylalanine ring in Arpeggio, whereas we take the centroid of those carbon atoms. The example case involving 3,5‐diiodotyrosine (Figure 3d) is not available at the PDBe ligand page, as the modified amino acid is not treated as a ligand, and no ligand page is created.

Additionally, interactions between halogen atoms and water molecules could be investigated further. While visually inspecting hundreds of example cases, we observed false positives where the halogen bond with a water molecule would be more likely than the acceptor we found while datamining. These should be considered in future pipelines. Note that with water molecules, there comes an extra complication, as the location of the free lone pairs depends on the hydrogen bonding network and is not always clear.

To further investigate C‐X···Π halogen bonds, the distance from the halogen perpendicular to the π‐system of the acceptor is worth calculating and including as a geometric parameter (Riley & Tran, 2017). Another option could be to use a θ2‐equivalent angle between the halogen atom, the centroid of the π‐system, and one carbon atom in the π‐system, which has an ideal value around 90°. This should ease the filtering of halogen‐acceptor pairs into actual halogen bonds wherein the σ‐hole of the halogen is positioned perpendicular to the π‐system. Also, halogen bonds can be formed with the π‐system of peptide bonds (Auffinger et al., 2004). Identifying such interactions in PDB‐REDO structure models would add an extra halogen bond type to the dataset that we have not examined here.

The availability of more accurate or higher‐resolution data will allow for more critical filtering, which will allow us to distinguish between a genuine halogen bond and “a halogen in proximity of a possible acceptor atom” more accurately. To add more observations and thereby increase the dataset size, it becomes more feasible to enrich the halogen bond types with specific distributions, for example, halogen bonds including non‐canonical amino acids or nucleotides, and to define distinct classes of halogen‐acceptor pairs like halogen bonds involving hydroxylic or carboxylic oxygen atoms. This can be particularly effective for fluorine and chlorine, which, as we have demonstrated, are less straightforward to identify with the current data. Besides these optimizations with respect to defining specific types of halogen‐acceptor pairs, more high‐quality data could contribute to the differentiation between a genuine halogen bond and “a halogen in proximity of a possible acceptor” as improving the quality of halogen bonds that are used as reference in HalBS calculations will probably result in more narrow distributions.

A valuable source of potential high‐resolution ligand‐protein halogen bonds can be crystallographic fragment screening data. Typically, the protein‐fragment complexes obtained in such screens are of high resolution (Maveyraud & Mourey, 2020). Furthermore, fragment libraries used in such screens typically hold many halogen‐containing compounds, albeit more fluorine and chlorine than bromine and iodine atoms. Overcoming data archiving issues (Erlanson et al., 2025; Jaskolski et al., 2022; Perrakis, 2025; Weiss et al., 2022) could allow valuable data from hundreds or even thousands of ligand‐protein complexes obtained from fragment screening to enrich halogen bond observations and hereby ease the definition of validation targets. Furthermore, relevant data will hopefully emerge through the OpenBind initiative that aims to generate publicly available massive, high‐quality, fit‐for‐purpose protein‐ligand structure and affinity datasets (https://openbind.uk/).

## 4 CONCLUSION

Halogen bonding interactions between ligands and proteins are important for drug design. Nevertheless, in most macromolecular structure validation pipelines, halogen bonds are not considered. Here, we identified halogen bonds between ligands and proteins in PDB‐REDO structure models and introduced a validation metric for halogen bonds: HalBS. We use HalBS to annotate halogen bonds adopting preferred or allowed geometry and flag outliers that should be examined critically. This approach is integrated into the PDB‐REDO pipeline and can be used in other ligand validation pipelines. Hereby, we increase awareness of this interaction but also realize that our approach only describes a limited selection of halogen bond types. We expect that by accumulating (high quality) data of halogen bonds in ligand‐protein complexes, it could become possible to determine validation targets with ideal values and corresponding standard deviations for each halogen‐acceptor pair.

## 5 METHODS

### 5.1 Data mining

Compound IDs of halogen‐containing compounds were mined from the Chemical Component Dictionary (CCD, (Westbrook et al., 2015)) of the Protein Data Bank (Burley et al., 2019) and stored with their type and the name(s) of the halogen atom(s) in the molecule. Compounds in which the halogen atom is not connected to a carbon atom, for example, in halogen‐metal complexes and halogen acids, and the single ion halogens (F–, Cl–, Br–, I–) were removed from this list to focus on organic halogen bonds. The identifiers of excluded halogen‐containing compounds are: F, CL, BR, IOD, 08T, 0JC, 0OD, 0TE, 202, 2T8, 4IR, 4KV, 5LN, 61C, 61D, 6BP, 6O0, 73M, 7GE, 8TH, 8TR, 8WV, 9QB, 9RU, 9TH, A9J, AF3, ALF, BE7, BEF, BF2, BF4, BFD, BPT, C2C, C7P, CFO, CPT, CUL, D0X, D7Z, DAA, DAE, DAQ, E3D, E5O, ELJ, F6Q, F7T, FPO, HG2, HGI, I2I, I3M, I83, J0K, J0N, JR3, KQB, KYS, KYT, LCO, LCP, LN8, MF4, MGF, MNQ, N2N, N2R, N2S, N2W, NG8, NMQ, NXC, O1N, ONP, ORS, OS1, OT1, P3C, PC4, PCL, PEJ, PNQ, PT7, QLT, R1N, RAX, RBN, RHE, RSW, RU0, RU7, RUD, RUH, SFL, SRX, SVP, SXC, TBR, TPT, U0J, VKZ, VL2, YPT, YXX, YXZ, ZN0, ZN5, ZN6, ZN7, ZN8, ZN9, ZPT.

### 5.2 Defining halogen bonds in PDB‐REDO structure models

For each of the PDB‐REDO (Joosten et al., 2009) structure models with at least one halogen‐containing compound, the halogen atoms were mined and stored with their possible acceptor atoms. An acceptor atom is an oxygen (O), nitrogen (N), sulfur (S), phosphorus (P), or selenium (Se) atom in an organic compound within 4 Å of the halogen atom itself. For each halogen‐acceptor pair, the distance between the two atoms and their Van der Waals overlap, as well as the θ1 and θ2 angles, were calculated. The Van der Waals radii of the atoms were taken from https://en.m.wikipedia.org/wiki/Atomic_radii_of_the_elements_(data_page) (Bondi, 1964; Clementi et al., 1967; Mantina et al., 2009; Pyykkö et al., 2005; Slater, 1964). Possible halogen bonds to water were excluded as θ2 angles could not be calculated.

Similarly, as for C–X··Y halogen bonds, we mined halogen bonds between a halogen atom and a π‐system found in either phenylalanine (PHE), tyrosine (TYR), histidine (HIS), or tryptophan (TRP) to find halogen‐π bonds within protein‐ligand complexes. Such an interaction was assigned when the distance between the halogen atom and the centroid of the π‐system was within 6 Å. The centroid of the π‐system was also used to calculate the θ1 angle for the interaction. For the halogen‐π interactions, θ2 and the Van der Waals overlap cannot be calculated.

When a halogen atom had multiple possible acceptors, the acceptor with the θ1 angle closest to 180° was selected for further analyses to get a single acceptor for each halogen atom. Halogen‐acceptor pairs with a θ1 angle <90° were removed from the data, as such angles would render the σ‐hole unreachable (Figure 1).

### 5.3 Mining metadata

For each structure model in which we found a halogen bond, the resolution of the X‐ray data, the R‐factor, the R‐free, average B‐factor, and PDB‐REDO version were stored. Finally, the real space correlation coefficient (RSCC, (Jones et al., 1991)), as calculated by density‐fitness (van Beusekom et al., 2019), was mined for both the halogen‐containing compound as well as the compound containing the acceptor.

### 5.4 Software

Mining of the halogen bonds was done using libcif++ (Hekkelman, 2024a) and libpdb‐redo (Hekkelman, 2024b). The obtained datasets were loaded using pandas2.1.4 (The pandas development team, 2020), plots were made using Seaborn0.13.2 (Waskom, 2021), all in Python3.12.1.

## AUTHOR CONTRIBUTIONS

**Ida de Vries:** Software; data curation; supervision; writing – original draft; methodology; investigation; conceptualization. **Georgia Tsiompanaki:** Investigation. **Anastassis Perrakis:** Supervision; writing – review and editing; funding acquisition. **Robbie P. Joosten:** Conceptualization; methodology; software; supervision; writing – review and editing.

## FUNDING INFORMATION

This project was funded by the Horizon2020 EC projects iNEXT‐Discovery (871037) and FragmentScreen (101094131), and by an institutional grant of the Dutch Cancer Society and the Dutch Ministry of Health, Welfare and Sport. We also thank Oncode Institute and CCP4 for their financial support and Pantelis G. Bagos for fruitful discussions on the data analysis.

## Supporting information


**DATA S1.**. Supplemental material figures and tables referenced in this manuscript are available in PDF format.

## Data Availability

Reference code for the implementation of HalBS score calculations in PDB‐REDO is available at https://github.com/PDB-REDO/HalBS.
