# Supplementary material for: Halogen bonds between ligands and proteins: Can we use them in validation?
Source: Protein Sci. 2025 Oct 11;34(11):e70321. doi: 10.1002/pro.70321 (PMC12514845; doi:10.1002/pro.70321)
Supplement: Supplementary file 1 — DATA S1.. Supplemental material figures and tables referenced in this manuscript are available in PDF format. [file PRO-34-e70321-s001.pdf]

Supplemental information

Supplemental Figure S1 Overall distributions of the halogen bonds in PDB-REDO, from left to right: Resolution of the structures wherein halogen bonds were found, B-factors of halogen atoms and their acceptors, RSCC (real space correlation coefficient) of donor and acceptors residues. Bin-widths from left to right: 0.1,10,10,0.01,0.01. A) quality indicators for C-X· ·Y bonds; B) quality indicators for C-X· · ·Π bonds.

A

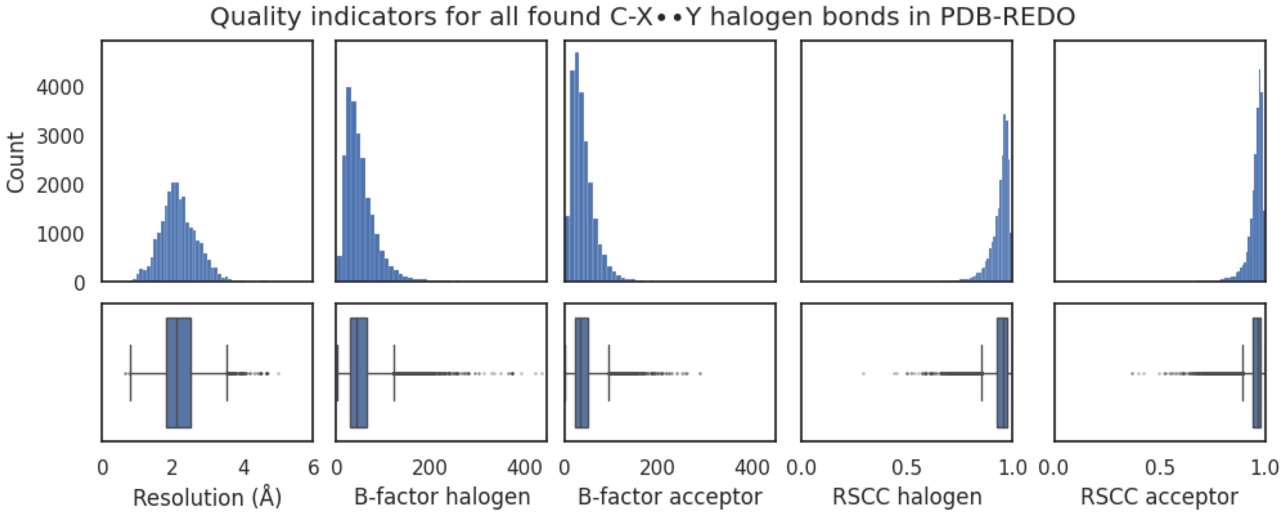

B

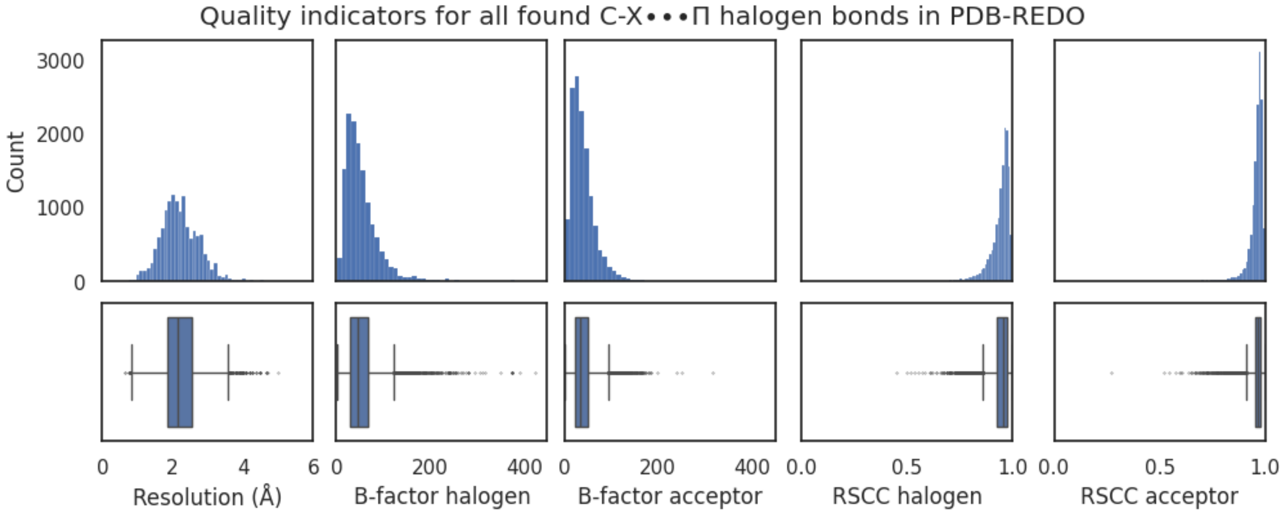

Supplemental Figure S2 Distributions of the quality indicators for the ligand-protein C-X· ·Y halogen bonds found in PDB-REDO after initial filtering. From left to right: Resolution of the structures wherein halogen bonds were found, B-factors of halogen atoms and their acceptors, RSCC (real space correlation coefficient) of the compounds containing the halogen or the acceptor atom. Bin-widths from left to right: 0.1,10,10,0.01,0.01. A) quality indicators for C-X· ·Y bonds with fluorine; B) quality indicators for C-X· ·Y bonds with chlorine; C) quality indicators for C-X· ·Y bonds with bromine; D) quality indicators for C-X· ·Y bonds with iodine.

A

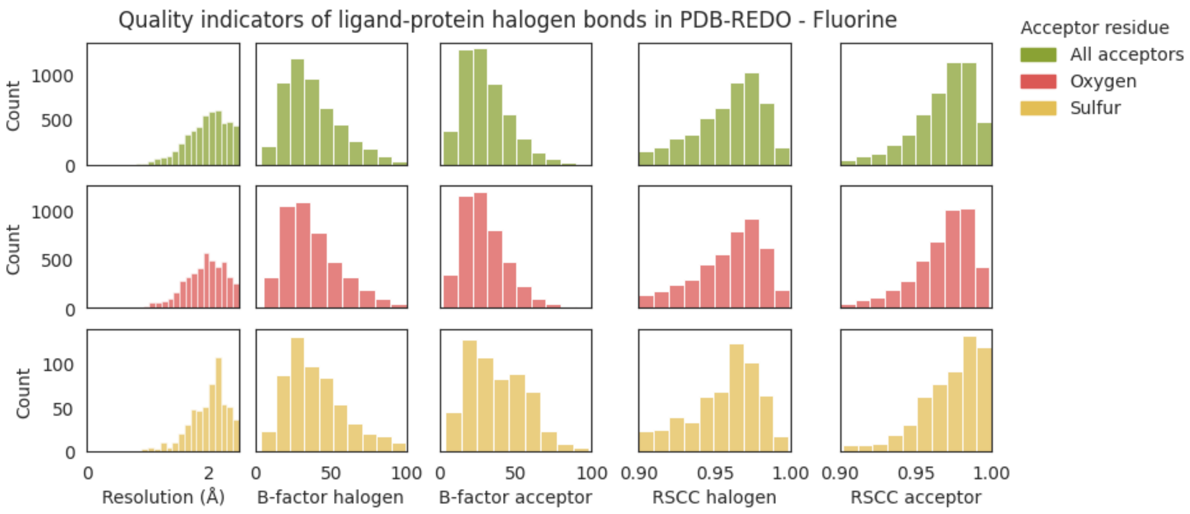

B

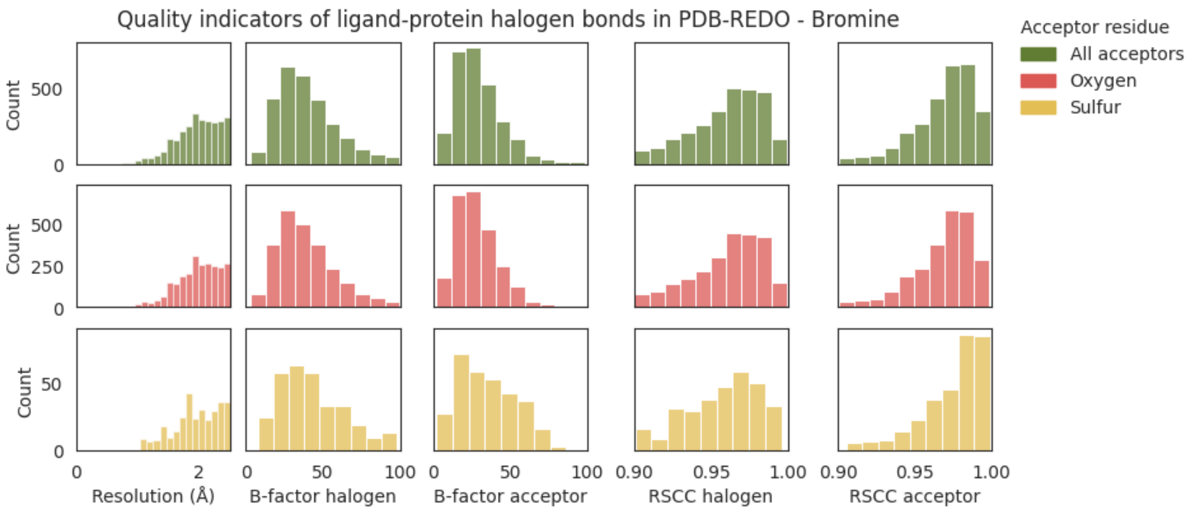

C

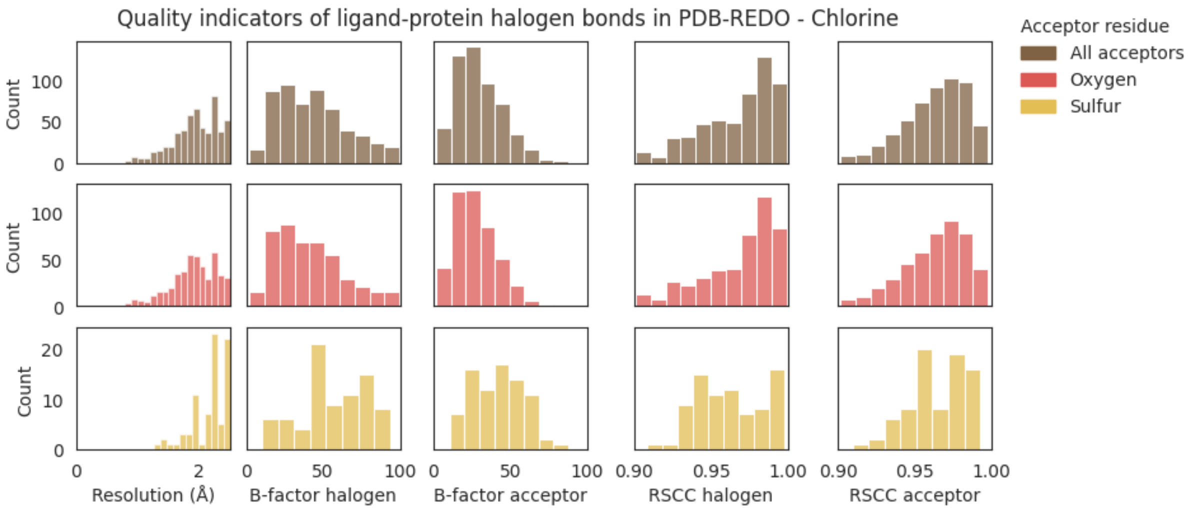

Quality indicators of ligand-protein halogen bonds in PDB-REDO - Chlorine
Acceptor residue
All acceptors
Oxygen
Sulfur
Count
Resolution (Å)
B-factor halogen
B-factor acceptor
RSCC halogen
RSCC acceptor

D

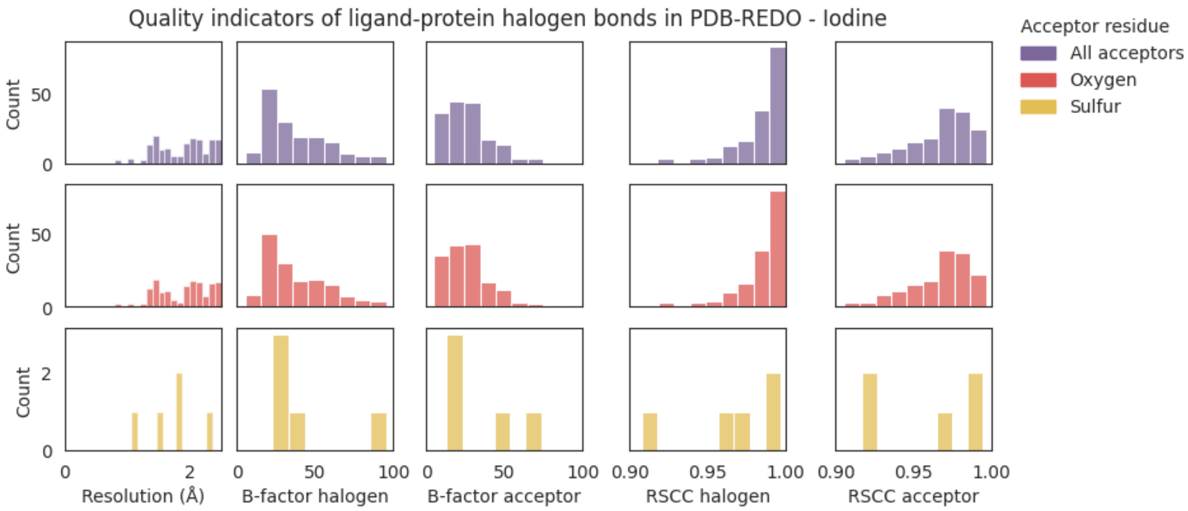

Quality indicators of ligand-protein halogen bonds in PDB-REDO - Iodine
Acceptor residue
All acceptors
Oxygen
Sulfur
Count
Resolution (Å)
B-factor halogen
B-factor acceptor
RSCC halogen
RSCC acceptor

Supplemental Figure S3 Distributions of the quality indicators for the ligand-protein C-X···Π halogen bonds found in PDB-REDO after initial filtering. From left to right: Resolution of the structures wherein halogen bonds were found, B-factors of halogen atoms and their acceptors, RSCC (real space correlation coefficient) of the donor and acceptor residues. Bin-widths from left to right: 0.1,10,10,0.01,0.01. A) quality indicators for C-X···Π bonds with fluorine; B) quality indicators for C-X···Π bonds with chlorine; C) quality indicators for C-X···Π bonds with bromine; D) quality indicators for C-X···Π bonds with iodine.

A

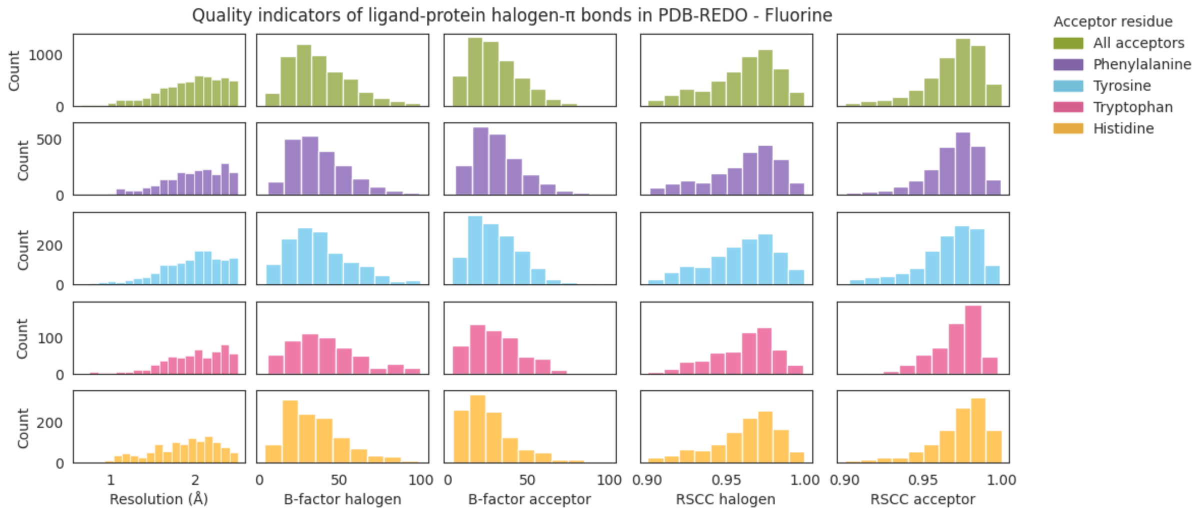

B

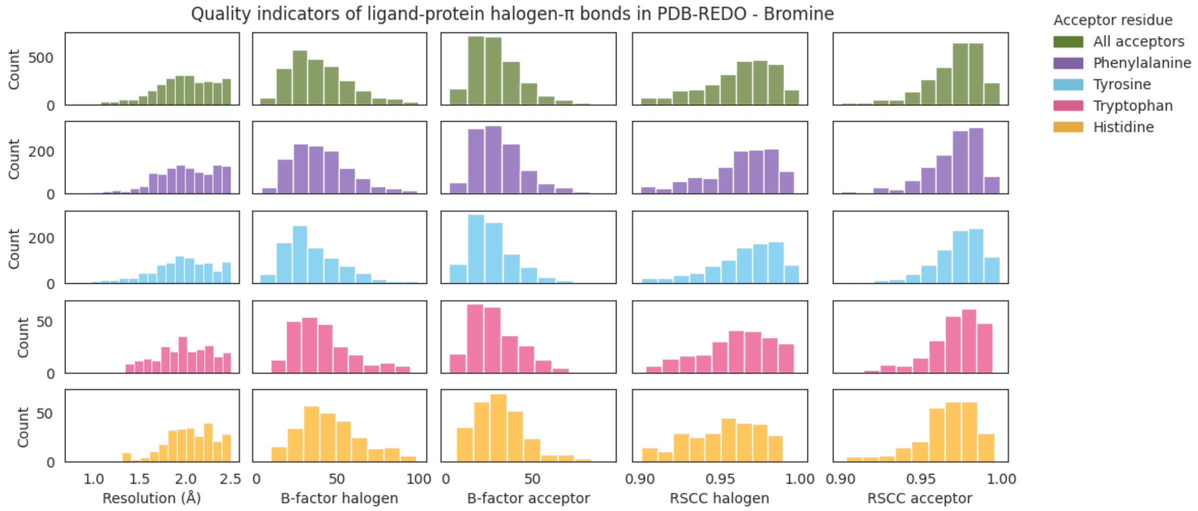

C

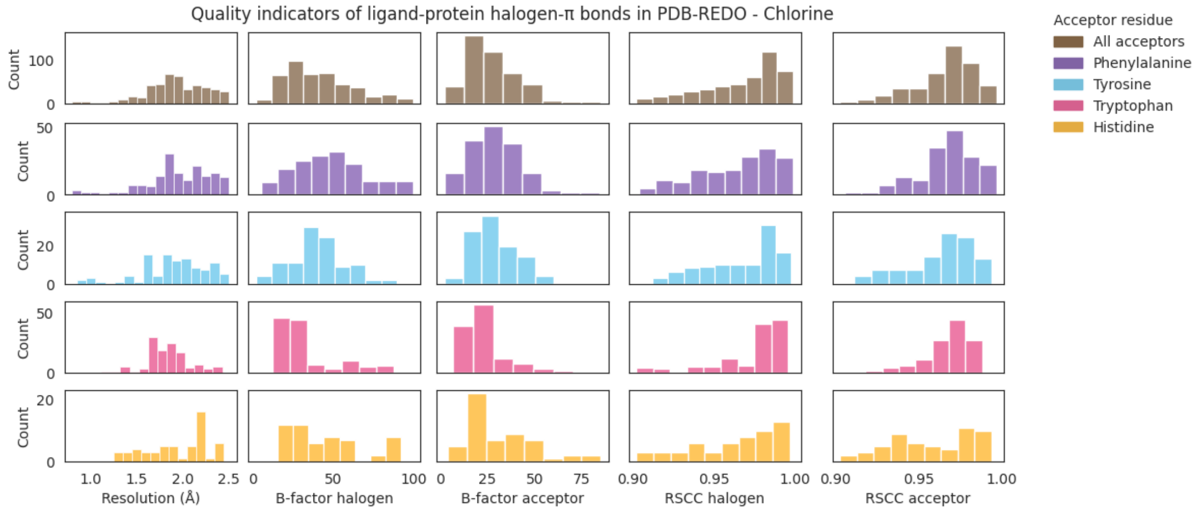

Quality indicators of ligand-protein halogen-π bonds in PDB-REDO - Chlorine
Acceptor residue
All acceptors
Phenylalanine
Tyrosine
Tryptophan
Histidine
Count
Resolution (Å)
B-factor halogen
B-factor acceptor
RSCC halogen
RSCC acceptor

D

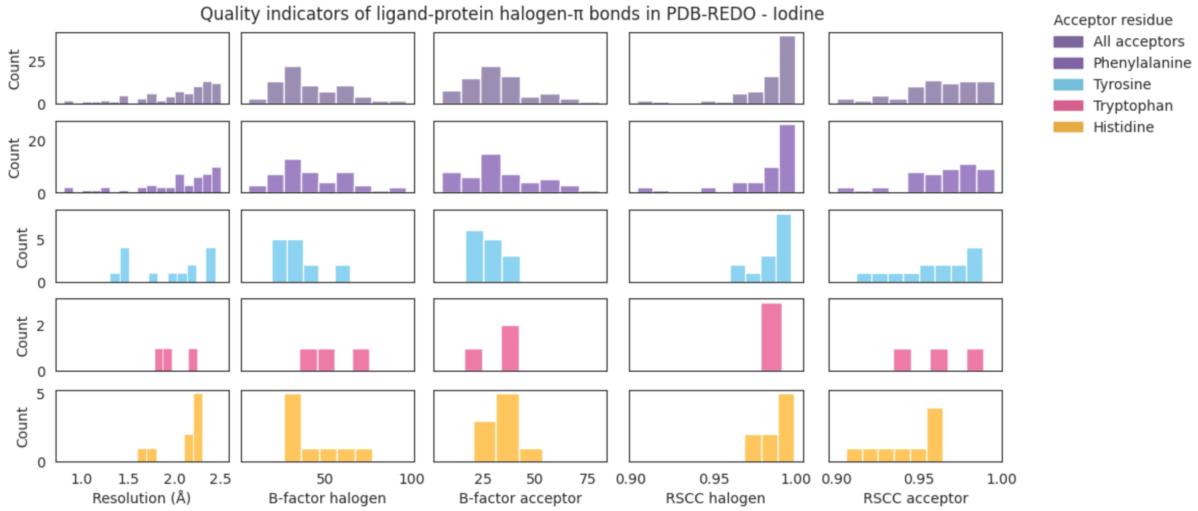

Quality indicators of ligand-protein halogen-π bonds in PDB-REDO - Iodine
Acceptor residue
All acceptors
Phenylalanine
Tyrosine
Tryptophan
Histidine
Count
Resolution (Å)
B-factor halogen
B-factor acceptor
RSCC halogen
RSCC acceptor

Supplemental Figure S4 Distributions of the geometric parameters for the ligand-protein C-X··Y halogen bonds found in PDB-REDO. From top to bottom: halogen bonds containing fluorine, chlorine, bromine and iodine, respectively. From left to right: All halogen bonds, halogen bonds with acceptor O-C, N-C, S-C, respectively. Y-axis show counts. A) Distribution of distances, bin-width 0.1A. B) Distribution of van-der-Waals overlap, bin-width 0.1Å. C) Distribution of θ1 angles, bin-width 5˚. D) Distribution of θ1 angles, bin-width 5˚.

A

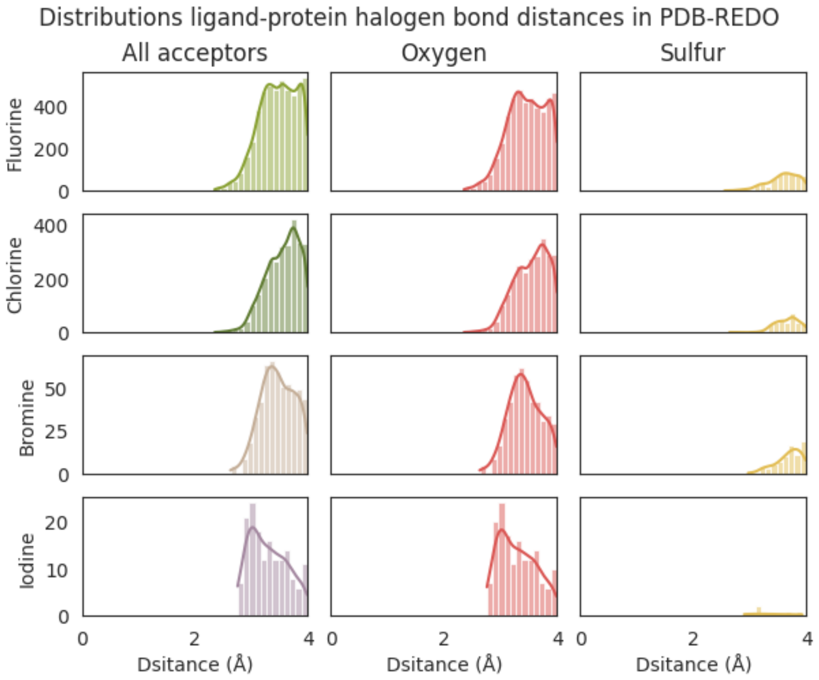

B

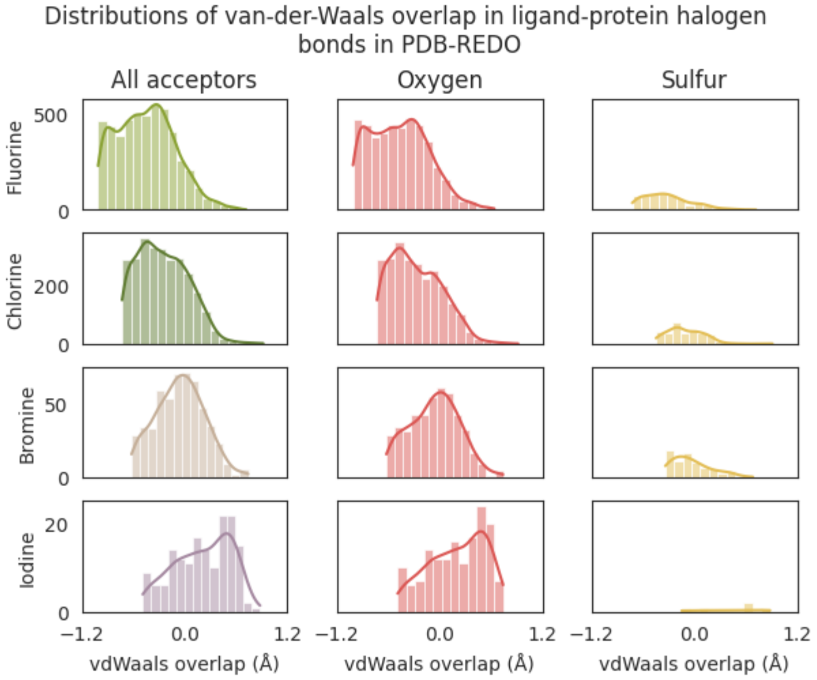

C

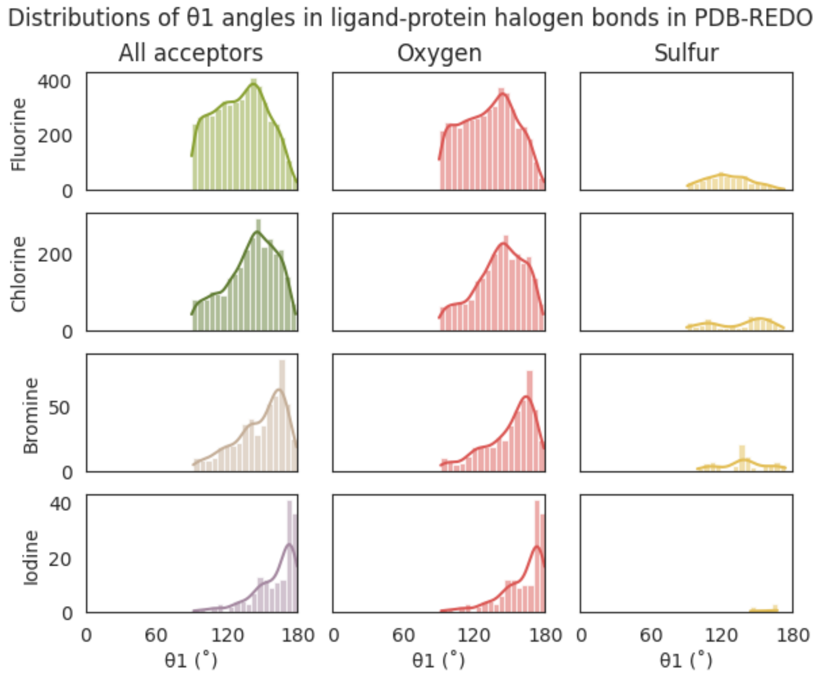
Distributions of θ1 angles in ligand-protein halogen bonds in PDB-REDO
All acceptors
Oxygen
Sulfur
Fluorine
Chlorine
Bromine
Iodine
400
200
0
50
40
20
0 60 120 180
θ1 (°)

D

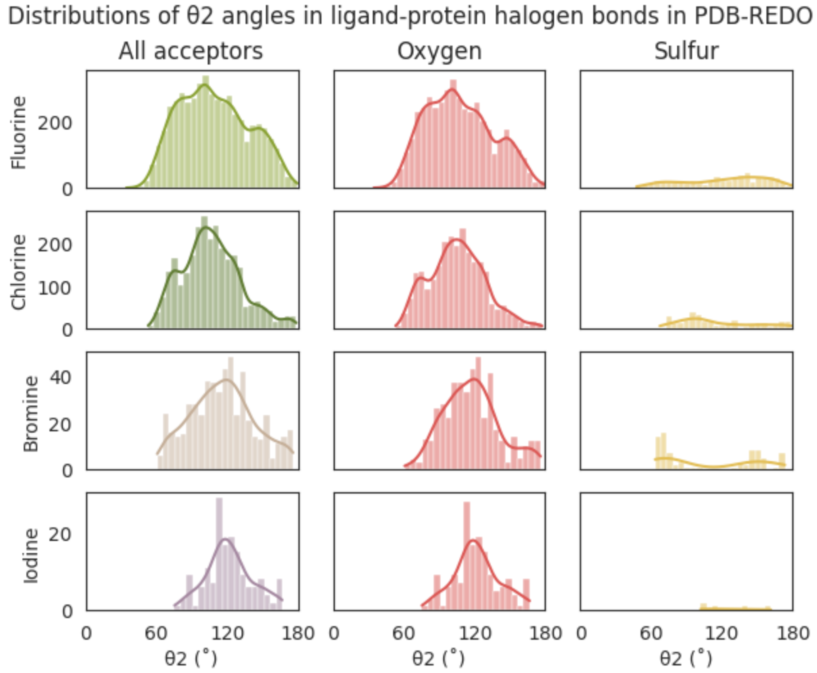
Distributions of θ2 angles in ligand-protein halogen bonds in PDB-REDO
All acceptors
Oxygen
Sulfur
Fluorine
Chlorine
Bromine
Iodine
200
100
0
40
20
0 60 120 180
θ2 (°)

Supplemental Figure S5 Distributions of the geometric parameters for the ligand-protein C-X···Π halogen bonds found in PDB-REDO. From top to bottom: halogen bonds containing fluorine, chlorine, bromine and iodine, respectively. From left to right: All halogen bonds, halogen bonds with acceptor phenylalanine, tyrosine, tryptophan and histidine, respectively. Y-axis show counts. A) Distribution of distances, bin-width 0.1Å. B) Distribution of θ1 angles, bin-width 5˚.

A

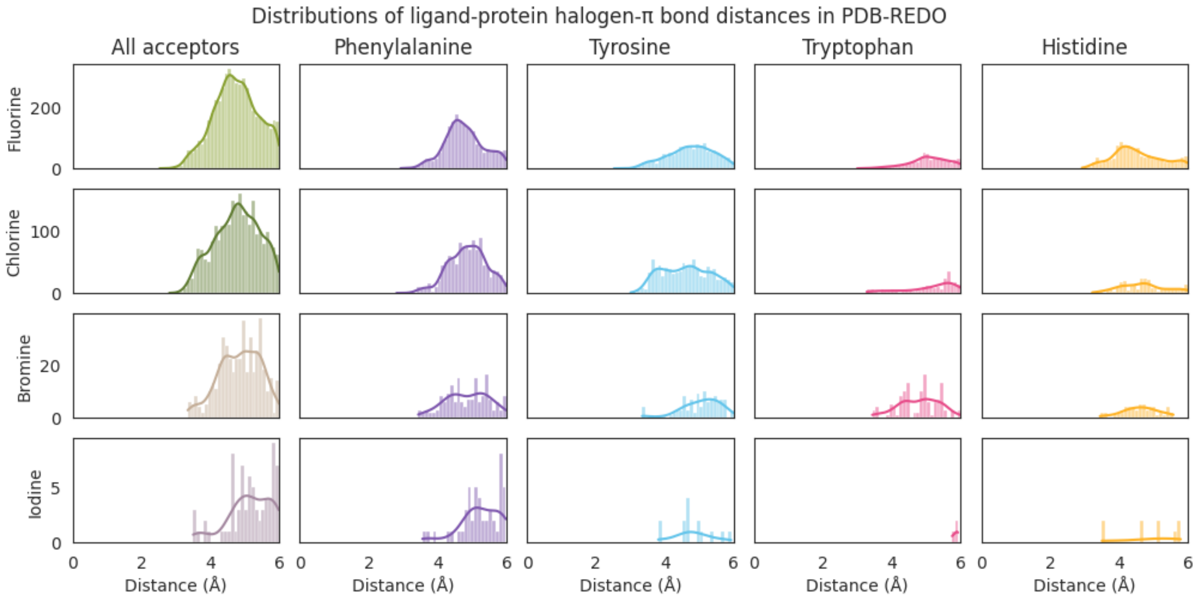

B

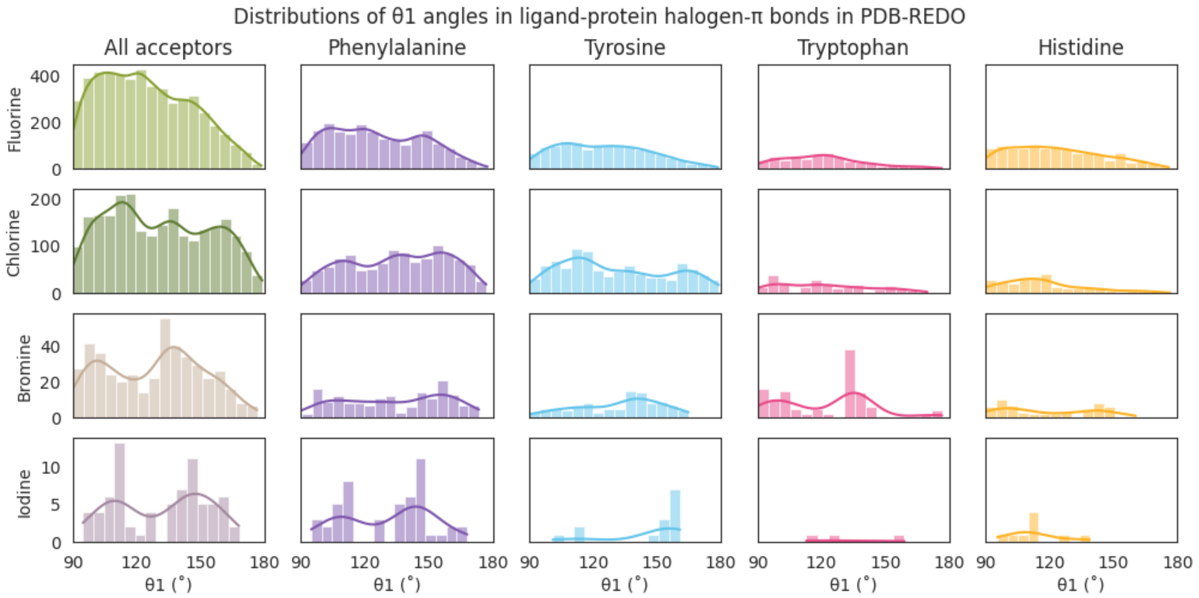

Supplemental Figure S6 Geometry of ligand-protein C-X··O halogen bonds found in PDB-REDO. For each halogen (from left to right: fluorine (light green), chlorine (dark green), bromine (brown), iodine (purple)) the overall distance, van-der-Waals overlap, θ1 and θ2 angles are shown on the left panel. In the panels on the right-hand side these values are split by acceptor oxygen type: Hydroxyl, Carboxyl and Carbonyl. Note that a positive value for the sum of the van-der-Waals radii means that the distance between halogen and acceptor atom is shorter than the van-der-Waals overlap. For clarity a strip plot is shown when there are 25 or fewer observations.

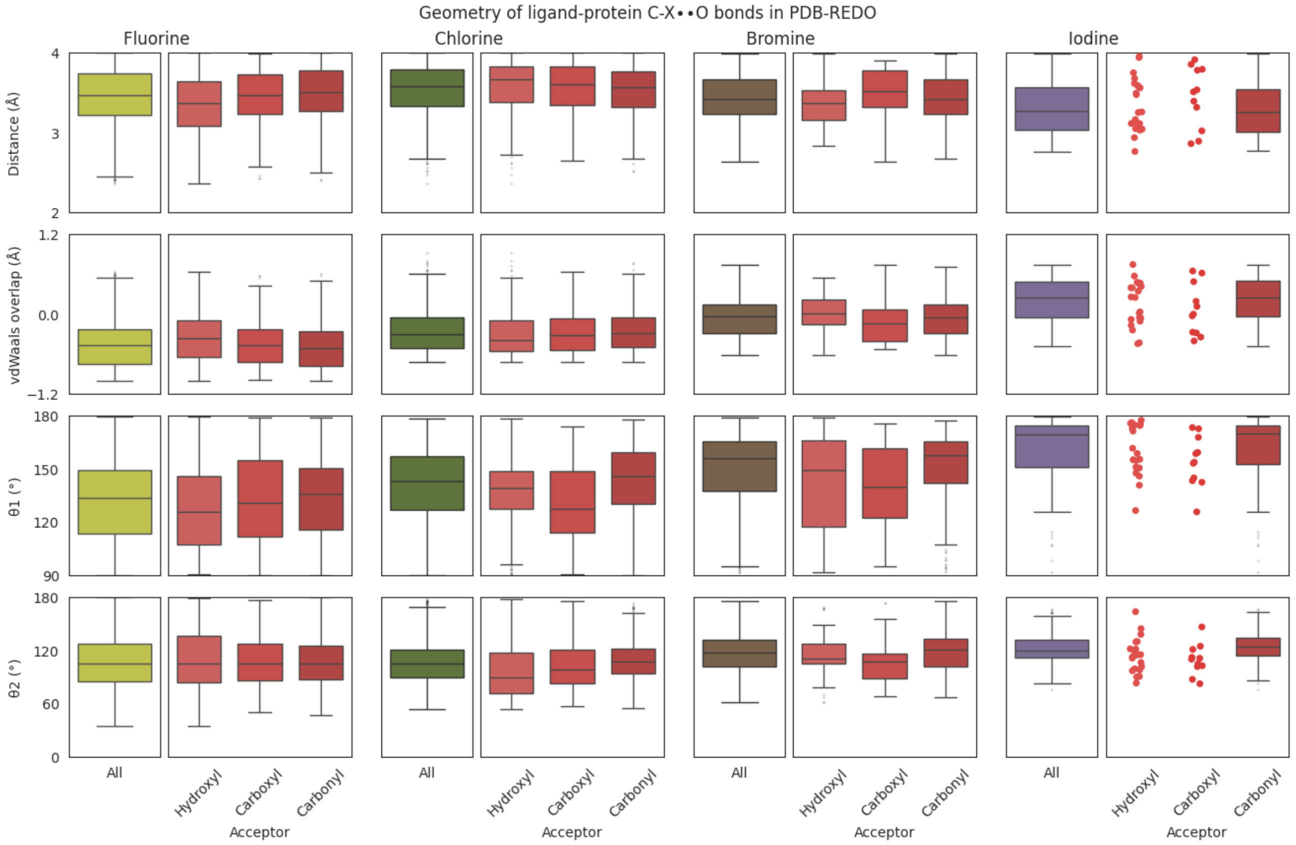

Supplemental Table S1 Number of observations of halogen-acceptor pairs for C-X··Y and C-X···Π bonds found in PDB-REDO structure models, with a crystallographic resolution of 2.5Å or better, a B-factor of 100 Å^2 or lower, and an RSCC equal to or greater than 0.9 for the donating and accepting residues. For C-X··Y bonds the acceptor is listed with its neighbouring atom, e.g. O-C is an oxygen atom as acceptor, which is connected to a carbon atom. For the accepting π-system is noted as the amino acid wherein it was found, e.g. Phe indicates that there is a halogen bond between the halogen and the π-system of the phenyl ring in the phenylalanine side chain.

	**Number of halogen-acceptor pairs**				
**Halogen / Acceptor**	**F**	**Cl**	**Br**	**I**	**Total**
	**C-X··Y halogen bonds**				
**O-C**	4640	2548	515	172	**7875**
**S-C**	571	314	80	11	**976**
**S-S**	3	4	0	1	**8**
**O-P**	3	1	1	0	**5**
**O-O**	1	0	1	0	**2**
**O-S**	1	0	0	0	**1**
**P-O**	2	0	0	0	**2**
**Se-C**	0	0	0	1	**1**
**Total**	**5221**	**2867**	**597**	**185**	**8870**
	**C-X···Π bonds**				
**Phe**	2146	170	1114	51	**3481**
**Tyr**	1389	125	908	20	**2442**
**His**	1216	63	258	12	**1549**
**Trp**	584	122	226	3	**935**
**Total**	**5335**	**2506**	**480**	**86**	**8407**

Supplemental Table S2 The compound types found in the CCD (Chemical Component Dictionary) of the halogen atom containing compounds for the observed halogen bonds.

	**Number of compounds**	
**Compound type**	**C-X· · Y** (Y = O-C or S-C)	**C-X· · · Π**
Non-polymer	8442	8096
l-peptide linking	153	64
d-peptidelinking	17	8
Peptide-like	13	18
DNA-linking	79	51
RNA-linking	25	21
l-saccharide	1	3
l-saccharide-alphalinking	8	5
l-saccharide-betalinking	0	3
d-saccharide	30	40
d-saccharide-alphalinking	46	64
d-saccharide-betalinking	37	34

Supplemental Table S3 Statistical values (number of observations, mean, median, Q1, Q3 and Median absolute deviation (MAD)) for the distance, Van-der-Waals overlap, θ1 and θ2 angles in C-X· ·Y halogen bonds corresponding to the boxplots shown in Figure 2A.

	F all	F· ·O	F· ·S	Cl all	Cl· ·O	C· ·S	Br all	Br· ·O	Br· ·S	I all	I· ·O	I· ·S
**Count**	4935	4381	554	2791	2479	312	536	456	80	161	156	5
**Distance (Å)**												
**Mean**	3.5	3.5	3.6	3.6	3.5	3.7	3.5	3.4	3.7	3.3	3.3	3.4
**Median**	3.5	3.5	3.6	3.6	3.6	3.7	3.5	3.4	3.7	3.3	3.3	3.2
**Q1**	3.2	3.2	3.4	3.4	3.3	3.5	3.3	3.2	3.6	3.0	3.0	3.1
**Q3**	3.8	3.7	3.8	3.8	3.8	3.8	3.7	3.7	3.9	3.6	3.6	3.7
**MAD**	0.3	0.3	0.2	0.2	0.2	0.1	0.2	0.2	0.2	0.3	0.3	0.3
**Van-der-Waals overlap (Å)**												
**Mean**	-0.5	-0.5	-0.3	-0.3	-0.3	-0.1	-0.1	-0.1	0.0	0.2	0.2	0.4
**Median**	-0.5	-0.5	-0.4	-0.3	-0.3	-0.1	-0.1	0	-0.1	0.2	0.2	0.6
**Q1**	-0.7	-0.8	-0.5	-0.5	-0.5	-0.3	-0.3	-0.3	-0.2	-0.1	-0.1	0.1
**Q3**	-0.2	-0.2	-0.2	0.0	-0.1	0.0	0.1	0.1	0.1	0.5	0.5	0.6
**MAD**	0.2	0.3	0.2	0.2	0.2	0.1	0.2	0.2	0.2	0.3	0.3	0.3
**θ1 (˚)**												
**Mean**	131.2	132.0	125.2	140.0	140.8	134.3	147.8	149.5	138.6	160.5	160.6	157.9
**Median**	132.1	133.4	123.5	143.2	143.2	143.2	154.0	155.7	137.7	169.2	169.6	164.4
**Q1**	112.9	113.1	111.1	125.4	126.8	110.4	135.9	137.2	131.2	150.4	150.8	148.4
**Q3**	148.5	149.5	138.5	156.9	157.3	155.4	165.0	165.4	154.3	174.0	174.4	165.6
**MAD**	17.6	17.9	14	15.2	15	16.6	13.6	12.2	13.2	6.9	7	2.5
**θ2 (˚)**												
**Mean**	109.4	107.8	122	106.7	105.5	116.3	116.4	117.9	107.9	122.0	122	123.9
**Median**	106.5	104.8	129.4	104.7	104.7	105.4	117.4	117.8	84.7	120.0	120.1	114.2
**Q1**	85.7	85.3	95.1	89.8	89.2	93.2	97.2	101.3	69.9	111.7	111.9	105.1
**Q3**	130.7	127.3	151.5	122.4	121.1	145	133.5	132.2	150.2	131.6	131.6	136.9
**MAD**	22.4	20.8	25.4	16.3	16.1	21.7	17.9	15.2	18.6	10.1	9.7	12.2

Supplemental Table S4 Statistical values (number of observations, mean, median, Q1, Q3 and Median absolute deviation (MAD)) for the distance, and θ1 angles in C-X···Π halogen bonds corresponding to the boxplots shown in Figure 2B. Cases with fewer than 5 observations are marked as 'NA' as no boxplot statistics can be calculated.

	**F all**	**F···Phe**	**F···Tyr**	**F···Trp**	**F···His**	**Cl all**	**Cl···Phe**	**Cl···Tyr**	**Cl···Trp**	**Cl···His**
**Count**	5099	2097	1328	539	1135	2477	1100	895	225	257
**Distance (Å)**										
**mean**	4.7	4.8	4.8	5.1	4.5	4.8	4.9	4.6	5.1	4.7
**median**	4.7	4.7	4.8	5.0	4.4	4.8	4.9	4.6	5.4	4.7
**Q1**	4.3	4.4	4.3	4.7	4.1	4.3	4.5	4.0	4.6	4.2
**Q3**	5.2	5.1	5.2	5.5	5.0	5.3	5.3	5.2	5.7	5.1
**MAD**	0.4	0.3	0.5	0.4	0.4	0.5	0.4	0.6	0.4	0.5
**θ1 (˚)**										
**mean**	124.6	125.7	125.3	120.7	123.4	130.6	135.4	130.6	121.6	117.6
**median**	122.2	123.1	123.8	119.2	121.1	129.3	137.0	126.2	119.0	114.8
**Q1**	106.8	107.5	107.4	105.6	105.9	110.7	115.0	110.7	100.6	103.3
**Q3**	141.0	144.4	141.1	131.6	139.0	151.2	155.0	151.8	137.1	127.4
**MAD**	16.8	18.1	16.7	13.1	16.5	19.9	19.3	19.2	18.1	11.6

	**Br all**	**Br···Phe**	**Br···Tyr**	**Br···Trp**	**Br···His**	**I all**	**I···Phe**	**I···Tyr**	**I···Trp**	**I···His**
**Count**	445	167	102	121	55	75	49	14	3	9
**Distance (Å)**										
**mean**	4.9	4.9	5	4.8	4.6	5.1	5.2	4.8	NA	4.9
**median**	4.9	4.9	5.1	4.9	4.6	5.1	5.2	4.7	NA	5.1
**Q1**	4.4	4.4	4.7	4.4	4.3	4.8	4.9	4.6	NA	4.6
**Q3**	5.4	5.4	5.4	5.3	5	5.7	5.8	5.0	NA	5.7
**MAD**	0.5	0.5	0.4	0.5	0.3	0.5	0.4	0.3	NA	0.5
**θ1 (˚)**										
**mean**	128.5	133.9	132.0	122.6	118.7	131.5	130.8	146.2	NA	112.7
**median**	133.4	134.1	137.0	133.2	112.5	136.8	136.8	154.0	NA	110.9
**Q1**	106.1	111.6	116.7	102.4	98.8	111.2	110.8	150.9	NA	106.4
**Q3**	145.4	155.8	145.8	135.3	141.2	147.6	146.1	157.8	NA	112.1
**MAD**	18.4	22.0	14.4	13.5	15.9	20.8	11.6	3.7	NA	4.5
